# Circular RNAs in Cell Cycle Regulation of Cancers

**DOI:** 10.3390/ijms25116094

**Published:** 2024-05-31

**Authors:** Pannathon Thamjamrassri, Chaiyaboot Ariyachet

**Affiliations:** 1 Department of Biochemistry, Faculty of Medicine, Chulalongkorn University, Bangkok 10330, Thailand; pannathon.tha@gmail.com; 2 Center of Excellence in Hepatitis and Liver Cancer, Faculty of Medicine, Chulalongkorn University, Bangkok 10330, Thailand; 3 Medical Biochemistry Program, Department of Biochemistry, Faculty of Medicine, Chulalongkorn University, Bangkok 10330, Thailand

**Keywords:** circRNAs, cancers, cell cycle regulators, oncogenic circRNAs, antitumor circRNAs

## Abstract

Cancer has been one of the most problematic health issues globally. Typically, all cancers share a common characteristic or cancer hallmark, such as sustaining cell proliferation, evading growth suppressors, and enabling replicative immortality. Indeed, cell cycle regulation in cancer is often found to be dysregulated, leading to an increase in aggressiveness. These dysregulations are partly due to the aberrant cellular signaling pathway. In recent years, circular RNAs (circRNAs) have been widely studied and classified as one of the regulators in various cancers. Numerous studies have reported that circRNAs antagonize or promote cancer progression through the modulation of cell cycle regulators or their associated signaling pathways, directly or indirectly. Mostly, circRNAs are known to act as microRNA (miRNA) sponges. However, they also hold additional mechanisms for regulating cellular activity, including protein binding, RNA-binding protein (RBP) recruitment, and protein translation. This review will discuss the current knowledge of how circRNAs regulate cell cycle-related proteins through the abovementioned mechanisms in different cancers.

## 1. Introduction

In 2023, the United States reported that the number of new cancer cases reached 1,958,310, while the number of casualties was estimated at 609,820. Lung and colorectum cancers contribute to the highest number of cancer-related deaths in both men and women [1]. Many internal biological factors have been identified to drive cancer progression, such as oncogenes, mutations in cell cycle-regulated genes, and aberrations in signaling pathways [2,3]. It was shown that mutations in p53, a cell cycle suppressor gene, occurred in 60% of cancer cases [2]. Furthermore, the mutated site in p53 usually occurs in the DNA-binding domain, thus disabling it from interacting with DNA [4]. Amplification of cell cycle-encoding genes, including cyclin D, cyclin E, and cyclin-dependent kinase 4 (CDK4), can also occur in certain types of cancer [5]. Additionally, several cancers were found to alter their signaling pathways that control the cell cycle [3,6]. Numerous proteins in the signaling pathway, such as PI3K/Akt and MAPK signaling, can be dysregulated due to pathway hyperactivation, allowing excessive cell proliferation [5].

Discovered several decades ago, circular RNAs (circRNAs) were once identified as junk [7]. However, high-throughput sequencing and bioinformatics analysis revealed that they are abundantly synthesized throughout all eukaryotes [8,9]. In fact, circRNAs play an important role in many cellular and physiological processes under normal conditions [10]. When dysregulated, several circRNAs are thus expected to participate in cancer development. Multiple studies also indicated that circRNAs are involved in abnormal signal transduction in cancer initiation, progression, metastasis, stemness, and resistance to therapy [11]. For example, overexpression of circZNF609 activates the Hedgehog signaling pathway by sponging miR-15a-5p and miR-15b-5p to enhance the stemness of hepatocellular carcinoma (HCC) cells [12]. CircPTK2 sponges miR-200a-3p to promote breast cancer tumorigenesis through the modulation of HIPPO/YAP signaling [13]. Interestingly, dysregulated circRNAs may indirectly influence the alteration of cell cycle regulator expression via controlling signaling pathways. To illustrate, circSHPRH sponges multiple miRNA targets, a process which results in the inhibition of JAK/STAT signaling and a reduction in cyclin-dependent kinase (CDK) 4, and -6 expression [14]. However, the signaling pathway that circRNAs mediate to control cell cycle regulator expression in many studies is still left unexplored and certainly requires more attention.

This review will focus on the influence of circRNAs on cell cycle-related protein expression in different types of cancer and associated signaling pathways.

## 2. Circular RNA and Biogenesis

**Figure 1:**
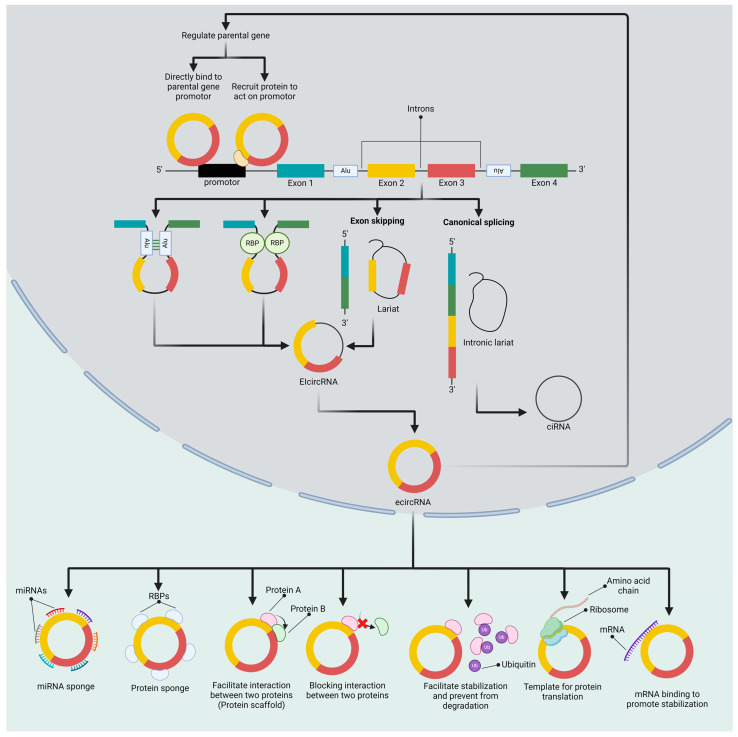
CircRNA biogenesis and its functions. CircRNA is a product of backsplicing, which begins with the joining of the downstream splice-donor site and the upstream splice-acceptor site, forming a circular loop-like structure. *Alu* or RNA-binding proteins (RBPs) can stimulate this interaction. The current circular loop-like structure, EIcircRNA, can also be generated via an exon-skipping event, and it might proceed through an internal splicing event to eliminate the intron, resulting in an ecircRNA. Moreover, canonical splicing can generate the other type of circRNA, ciRNA, which is entirely composed of introns. CircRNAs might be imported to the cytoplasm, where they primarily function. Mostly, circRNAs possess the ability to bind to miRNAs to regulate gene expression. CircRNA was recently found to be a template for protein translation or to establish an interaction with proteins. It can act like a protein sponge, which might later facilitate or inhibit the interaction between two different proteins. Utilizing protein sponge activity, some proteins avoid ubiquitin-mediated degradation by binding to circRNAs. Remarkably, some circRNAs have also been discovered to regulate their parental genes through various mechanisms, such as directly binding to the promotor or recruiting proteins to mediate gene expression.

CircRNA is a single-strand noncoding RNA with a covalently closed structure, lacking 3′ poly(A) tail modification and 5′ cap [15] (Figure 1). It is produced from a pre-mRNA splicing event called backsplicing, in which the downstream splice-donor site is closely brought to the upstream splice-acceptor site in a loop-like manner [16]. Invert repeat sequences, such as *Alu*, and dimerized *trans*-acting RNA-binding proteins (RBPs) are known to mediate this loop formation [17]. These RBPs include protein quaking (HQI), fused in sarcoma (FUS), and nuclear factor 90/110 (NF90/NF110) [18,19]. Moreover, NF90/110 was found to promote the stabilization of intronic RNA pairs, enhancing circRNA biogenesis [20]. The combination of multiple heterogeneous nuclear ribonucleoproteins (hnRNPs) and serine-arginine (SR)-rich proteins can also drive circRNA formation [21]. In contrast, the adenosine deaminase (ADAR) enzyme and ATP-dependent RNA helicase A (DHX9) interfere with circRNA biogenesis by blocking the complementary base pairing between invert repeat sequences [22,23]. However, backsplicing is less preferred than canonical splicing due to its low efficiency [24].

In addition to backsplicing, circRNAs can be generated from exon skipping in an alternative splicing event to form looped structures containing both exons and introns. This structure is called lariat, which can further undergo circularization and become exon–intron circRNA (EIcircRNA) or be processed by internal splicing to remove the intron, becoming exonic circRNA (ecircRNA) [25,26]. In addition, the canonical splicing byproduct, intronic lariat, can also escape from debranching and eventually circularize into circular intronic RNA (ciRNA) [27]. Interestingly, some studies have revealed that the fused gene in some cancers can give rise to circRNAs. For example, F-circBA1 is a product derived from a fusion of the BCR and ABL genes in chronic myeloid leukemia [28]. F-circEA1 in non-small-cell lung cancer (NSCLC) also results from the fusion of the EML4 and ALK1 genes [29].

## 3. Functions of circRNAs

The majority of circRNAs are proposed to act as miRNA sponges or decoys. Although miRNA was found to share a miRNA-binding element (MRE) not only with circRNA but also with other non-coding RNAs such as long-noncoding RNAs and pseudogenic RNAs. Competing endogenous RNAs (ceRNAs) were termed to describe the non-coding RNAs that are capable of binding to MRE. Therefore, it can be implied that the interaction between circRNA and its target miRNA might fall under the ‘multiple-to-multiple’ model, which is composed of a complex regulatory network involving ceRNAs [30,31]. Factors known to influence ceRNA activity are its abundance, subcellular localization of ceRNAs and miRNAs, miRNA-ceRNA affinity, and interaction with RNA-binding proteins (RBPs) [32,33,34]. However, more study is needed to completely clarify ceRNA activity, which might deeply affect the action of circRNAs on miRNAs.

In addition to being miRNA sponges, some circRNAs have been confirmed to encode a protein with biological function [35]. More evidence also revealed that they can regulate the expression of the parental gene by interacting directly with the promoter or forming a protein complex. Indeed, circRNA is thus capable of recruiting RBPs, binding to proteins, or acting as a protein sponge [26].

Recent studies showed that circRNA plays an important role in several cellular activities via post-transcriptional regulation (Figure 1). Therefore, dysregulation in their expression could lead to abnormalities in many biological processes and might eventually lead to the development of disease, not only cancer. To illustrate, an upregulated circCHD2 promotes hepatic leukemia factor (HLF) expression to stimulate the activation of hepatic stellate cells, an essential driver of liver fibrosis [36,37].

### 3.1. Regulating the Transcription Activity of the Parental Gene

Interestingly, parental gene expression was found to be repressed by its transcribed circRNAs as they bind to the synthesis locus, forming an RNA-DNA hybrid called R-loop [38]. The interaction halts the parental gene transcription activity [38]. CircSEP3 and circSMARCA5 are known to mediate such functions [38,39]. EIcircRNA establishes the complex with U1 small nuclear ribonucleoprotein (U1 snRNP) and binds to the promotor to upregulate the expression of the parental gene. Similar to EIcircRNA, ciRNAs accumulate at their synthesis site and elongate polymerase III activity to increase parental gene expression [27].

### 3.2. RNA Binding

Other than regulating its parental gene, circRNA has been mostly described as miRNA sponges or miRNA decoys as they contain many miRNA-binding sites, protecting mRNA targets from degradation. Moreover, recent studies also revealed that they can bind to mRNA to directly modulate post-transcriptional regulation. To illustrate, circPAN3 reinforces the stability of interleukin-13 (IL-13) mRNA through direct interaction [40].

### 3.3. Protein Translation

N6-methyladenosine (m^6^A) is a type of RNA modification that can be controlled by readers, writers, and erasers. The presence of m^6^A in circRNA can lead to various regulation mechanisms, including circRNA metabolism, localization, degradation, biogenesis, and protein encoding [41,42,43,44,45]. YTH N6-methyladenosine RNA-binding protein F3 (YTHDF3) recognizes the m^6^A-modified start codon and interacts with eIF4G2 to initiate protein translation. Mett13/14 was found to promote this activity, whereas fat mass and obesity-associated protein (FTO) inhibit the translation [45]. With the presence of an internal ribosome entry site (IRES) in some circRNA, ribosomes can be recruited and begin translation [46]. Moreover, circZNF609 utilizes the combination of the abovementioned processes to translate proteins, as m^6^A modification enhances the efficiency of IRES-mediated translation [47]. However, the protein encoded from circRNA can act against or be analogous to that of its linear counterpart [48,49].

### 3.4. Protein Binding

Numerous studies have indicated the protein-binding function of circRNAs [15]. However, bioinformatics analysis revealed that circRNA features a lower RBP binding density compared to linear RNA, suggesting that many circRNAs cannot interact with proteins [50]. The binding of circRNA to proteins may result in a different action. CircRNA binds to two different proteins to facilitate their activity, acting as a scaffold. To illustrate, circFOXO3 recruits both p53 and mouse double minute 2 (MDM2), resulting in MDM2-mediated p53 ubiquitination and degradation [51]. Another example, circNFIX, binds to Y-box binding protein 1 (Ybx1) and Nedd4I, a ubiquitin ligase, to promote ubiquitination of Ybx1 by Nedd4I and trigger degradation [52]. In other circumstances, circRNA binds to one protein and inhibits it from interacting with the other protein that does not bind directly to circRNA. For example, circGSK3β interacts with its parental gene product, GSK3β, which further phosphorylates β-catenin. By binding to circRNA, GSK3β is therefore prevented from exerting its function to induce β-catenin degradation [53]. Similar to circGSK3β, polypyrimidine track-binding protein 1 (PTBP1) is targeted by circH19, and this interaction impedes PTBP1-mediated SREBP1 cleavage [54]. CircRNA can also yield a similar result by attracting both proteins, which were originally combined in order to function, and preventing their association [15]. Naturally, CCNB1 and CDK1 form a complex to initiate mitosis progression, including condensation of chromosomes, spindle pole assembly, and nuclear envelope breakdown [55,56]. CircCCNB1 abolishes their activity by binding to both CCNB1 and CDK1, impairing complex formation [57]. In addition, circRNA is also capable of recruiting RBPs, which regulate mRNA splicing, stability, and translation, thus forming the circRNA–protein–mRNA ternary complex. CircNSUN2 binds to IGF2BP2 and enhances its ability to stabilize HMGA2 mRNA [42]. CircPOK was also shown to possess a similar mechanism by recruiting interleukin enhancer binding factor 2/3 (ILF2/3). The complex formation allows ILF2/3 to stabilize its target, IL-6, and VEGF mRNA [58].

## 4. Cell Cycle and Regulators

The cell cycle is a biological process involving cellular growth and proliferation. It can be divided into four separate phases: G1, S, G2, and M phase or mitotic phase. G1, S, and G2 phases are included in interphase, while M phase can be divided into mitosis and cytokinesis [59,60]. Although cells can exit the cell cycle into a quiescent state (G0) under specific conditions [61]. The progression to the next phase is mainly regulated by CDK and cyclin [62].

During the G1 phase, CDK4 or CDK6 associates with cyclin D to form cyclin D–CDK4/6 complex, which partially phosphorylates retinoblastoma protein (RB) [63,64]. The RB phosphorylation triggers detachment between RB and its target, E2F, a transcriptional factor that facilitates early cell cycle gene expression [65]. E2F upregulates the protein levels of cyclin A, cyclin E, and c-Myc [66]. With the activation of c-Myc, CDK2 can bind to cyclin E to establish cyclin E–CDK2 complex and then fully phosphorylate RB, proceeding to the S phase [64]. In the S phase, cyclin A joins CDK2 to form cyclin A–CDK2 complex, which is essential in stimulating the expression of proteins involved in DNA synthesis [67]. After entering the G2 phase, cyclin B–CDK1 complex gradually increases throughout the phase from the beginning to the end, promoting cells to the M phase. However, cyclin B–CDK1 complex begins to decline slowly in the M phase, which in turn drives the completion of mitosis [68].

In order to be tightly controlled, cyclin or CDK are regulated by several cell cycle regulators, such as inhibitors of the CDK4 (INK4) family, which impede cell cycle progression at the G1 phase by competitively binding to CDK4 or CDK6. This prevents the association with D-type cyclins. The INK4 family contains p15^INK4b^ (Cdkn2b), p16^INK4a^ (Cdkn2a), p18^INK4c^ (Cdkn2c), and p19^INK4d^ (Cdkn2d). WEE1 kinase can also phosphorylate CDK1 and lead to its inactivation. Another well-known cell cycle inhibitor is the CIP/KIP protein family. Cdkn1a (p21^CIP1^), Cdkn1b (p27^KIP1^), and Cdkn1c (p57^KIP2^) are included in this protein family [69]. With the activation of p53, CIP/KIP could inhibit the kinase activity of CDK1, CDK2, and other cyclin–CDK complexes. The cell division cycle 25 (CDC25) protein family also participates in cell cycle regulation by dephosphorylating CDK, resulting in CDK activation. For instance, CDC25A dephosphorylates CDK2 in the cyclin A–CDK2 complex, while CDC25C removes phosphate from CDK1 in the cyclin A/B–CDK1 complex [63]. In addition, there are several cellular signaling pathways indirectly affecting the cell cycle such as the PI3K/Akt, Wnt/β-catenin, JAK/STAT, Hedgehog, and MAPK signaling pathways.

Cell cycle regulators can also be post-transcriptionally controlled via miRNA-mediated suppression. For example, the miR-15 cluster promotes cell cycle arrest by targeting multiple key cell cycle regulators, including CDK1, CDK2, CDK6, cyclin D1, cyclin D3, and cyclin E1 [70,71]. Similarly, CDK6 expression was also found to be repressed by miR-107 [72].

## 5. CircRNA and Cell Cycle Regulators in Cancer

**Figure 2:**
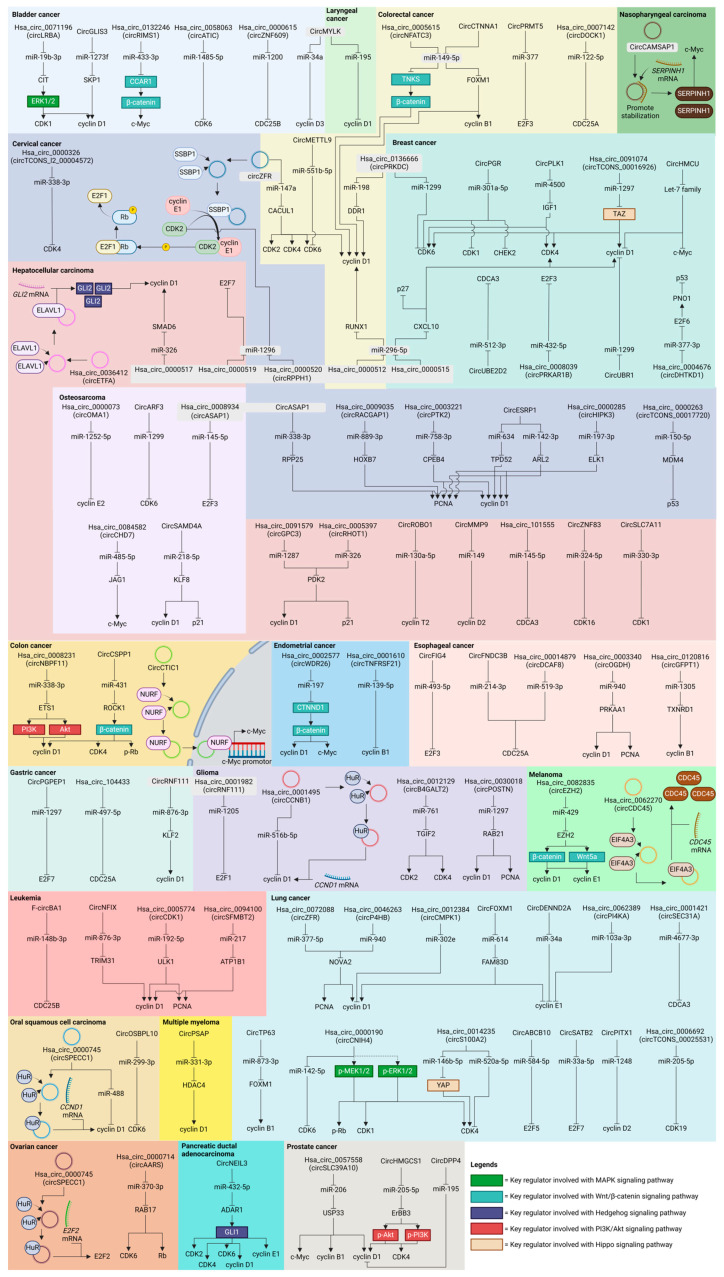
Oncogenic circRNAs and their path of action through various cell cycle regulators in bladder cancer, breast cancer, cervical cancer, colon cancer, colorectal cancer, endometrial cancer, esophageal cancer, gastric cancer, glioma, hepatocellular carcinoma, laryngeal cancer, leukemia, lung cancer, melanoma, multiple myeloma, nasopharyngeal carcinoma, oral squamous cell carcinoma, osteosarcoma, ovarian cancer, pancreatic ductal adenocarcinoma, and prostate cancer.

**Figure 3:**
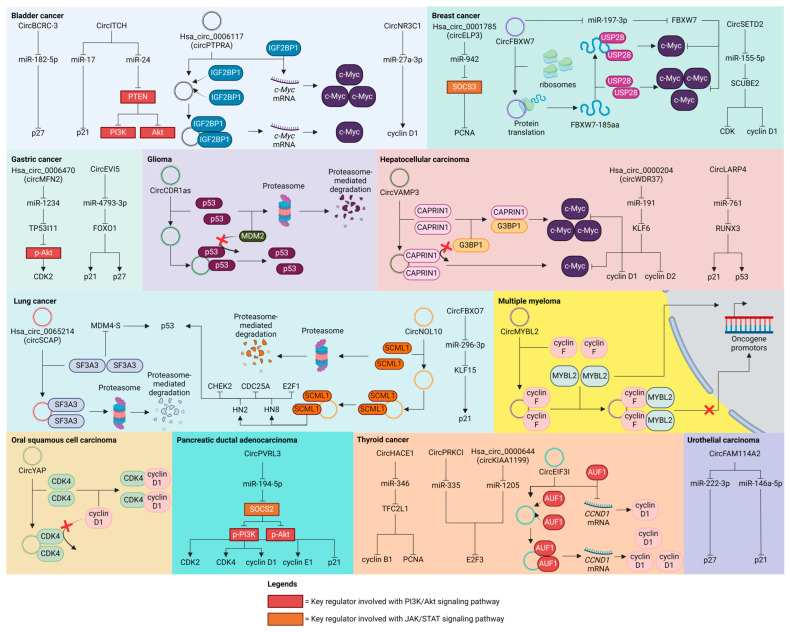
Tumor-suppressing circRNAs and their path of action through various cell cycle regulators in bladder cancer, breast cancer, gastric cancer, glioma, hepatocellular carcinoma, lung cancer, multiple myeloma, oral squamous cell carcinoma, pancreatic ductal adenocarcinoma, thyroid cancer, and urothelial carcinoma.

CircRNAs have become important mediators in the biology of cancer, deeply entwined with cell cycle regulation. Their carcinogenic potential is demonstrated in Figure 2, where specific circRNAs influence cell cycle regulators to cause abnormal cell proliferation. On the other hand, circRNAs with tumor-suppressive characteristics are shown in Figure 3, frequently through the inhibition of important cell cycle machinery components. Table 1 presents a thorough summary of the varied functions of circRNAs in different types of cancer, emphasizing their complex significance in carcinogenesis and their potential as targets.

### 5.1. Bladder Cancer

Numerous cellular signaling pathways contribute to the progression of the cell cycle, and certain circRNAs utilize these pathways to manipulate the behavior of cancer cells. For instance, it has been demonstrated that the mitogen-activated protein kinase (MAPK) signaling pathway positively regulates cyclin D1 expression and enhances the activation of the cyclin–CDK complex during the G1 phase [195]. CircLRBA, a circRNA that is naturally upregulated in bladder cancer tissue and cell lines, is known to aggravate the cell cycle of bladder cancer via MAPK signaling manipulation. Mechanistically, circLRBA sponges miR-19b-3p to enhance citron Rho-interacting serine/threonine kinase (CIT) expression. CIT suppresses the activation of phosphorylated ERK1/2, which is a part of the downstream signaling pathway of MAPK. This leads to an increase in the expression of CDK1 and cyclin D1, while the activity of p53 is suppressed [73]. In addition to the MAPK signaling pathway, the Wnt/β-catenin signaling pathway also plays an important role in driving the cell cycle. The evidence indicates that β-catenin induces the expression of c-Myc and cyclin D1, leading to the advancement of the G1 phase [196,197]. By utilizing Wnt/β-catenin signaling, circRIMS1 can reinforce cell cycle activity by sponging miR-443-3p [74]. By counteracting the inhibitory influence of miR-443-3p on its target, cell division cycle and apoptosis regulator 1 (CCAR1), which is recognized as a co-activator of β-catenin [198]. An increase in CCAR1 level thus activates the downstream cascade of the pathway and ultimately upregulates c-Myc, respectively [74].

Several circRNAs were known to directly promote cell cycle-related proteins, regardless of the cellular signaling pathway. For example, hsa_circ_0058063 (circATIC) positively regulates CDK6 by endogenously competing with miR-1485-5p, which inhibits *CDK6* mRNA translation. As a result, CDK6 expression is then enhanced due to the downregulation of miR-1485-5p [75]. Hsa_circ_0000615 (circZNF609) also sponges miR-1200 to increase its downstream target expression, CDC25B [76]. According to the prior study, CDC25B is required to stimulate the G2-M phase transition, and it also regulates checkpoint proteins in response to DNA damage [199]. Another example is that circMYLK can upregulate cyclin D3 expression by specifically targeting miR-34a. Once sequestrated by circMYLK, the downstream target of miR-34a, CCND3, can be more readily translated into cyclin D3 [77]. Recent research revealed that circGLIS3 can also support cell cycle progression by sponging miR-1273f, which targets S-phase kinase-associated protein (SKP1). The inhibition of miR-1273f leads to the activation of SKP1, which in turn stimulates the production of cyclin D1 and facilitates the transition from the G0/G1 phase to the S phase in the cell cycle [79].

Despite the majority of circRNAs that promote cell cycle progression, a few studies have also shown that some circRNAs have anti-cell cycle properties. Naturally downregulated in bladder cancer cell lines and tissue, circBCRC-3 was found to inhibit cell cycle progression by sponging miR-182-5p, thus upregulating its target expression, p27. The rise in p27 levels hinders the shift from G0/G1 to S phase [80]. Another essential signaling pathway involving the cell cycle is PI3K/Akt signaling. Multiple studies have indicated that PI3K/Akt signaling promotes cell cycle activity. To illustrate, Akt promotes the G1 progression by phosphorylating p21, which normally forms complexes with proliferating cell nuclear antigen (PCNA), cyclin, and CDK [200]. Phosphorylation of p21 in T145 residue leads to the dissociation of PCNA from the quaternary complex and a reduction in the binding affinity of p21 [200,201]. Free PCNA binds to DNA polymerase δ holoenzyme to promote DNA synthesis, while p21 decreases its inhibitory function on CDK2 [202]. CircITCH utilizes this signaling network to suppress bladder cancer proliferation by regulating multiple miRNAs, including miR-17 and miR-224. Notably, miR-17 specifically targets p21, while miR-224 specifically targets PTEN mRNA. The overexpression of circITCH thus results in the upregulation of p21 and PTEN, which is considered a key inhibitor of PI3K/Akt signaling [81,203]. Hsa_circ_0006117 (circPTPRA) impairs the G0/G1-S phase transition by interacting with insulin-like growth factor 2 binding protein 1 (IGF2BP1), an N^6^-methyladenosine (m^6^A) reader [82]. It is shown that IGF2BP1 can identify m^6^A-modified mRNAs and influence the stability and translation of the mRNA it targets [204]. Therefore, the interaction with circPTPRA blocks IGF2BP1 from recognizing m^6^A-modified mRNAs, including *c-Myc* mRNA, eventually suppressing the cell cycle [82]. Employing an uncommon molecular mechanism, circNR3C1 targets miR-27a-3p, which further binds to the 5′-UTR of *CCND1* to promote cyclin D1 expression. Therefore, the reduction in miR-27a-3p sequestered by circNR3C1 has a detrimental impact on *CCND1* translation, decreasing the cyclin D1 level and impeding the cell cycle [83].

### 5.2. Breast Cancer

Hsa_circ_0091074 (circTCONS_00016926) absorbs miR-1297, which is a repressor of transcriptional coactivator with PDZ-binding motif (TAZ). Given that TAZ is a downstream effector of the Hippo signaling pathway. Without the inhibition from miR-1297, TAZ is relieved from repression and induces the protein levels of cyclin D1, CDK4, and CDK6. However, circTCONS_00016926 is usually downregulated in breast cancer tissue and cell lines and does not exhibit cell cycle supportive activity in normal condition [84]. Naturally upregulated by the induction of estrogen, circPGR aggravates breast cancer progression by suppressing its target, miR-301a-5p, which partially binds to *CDK1*, *CDK6*, and checkpoint kinase 2 (*CHEK2*) mRNA. Thus, the decrease in miR-301a-5p level mediated by circPGR can lead to the upregulation of the translated products of the abovementioned mRNAs [85]. Interestingly, circHMCU was found to hold potential for binding miR-let7 family, which comprises of let-7a-5p, let-7c-5p, and let7-7f-5p. Therefore, their downstream target mRNAs: c-Myc; HMGA2; and cyclin D1, are then prevented from degradation. Furthermore, the expression of other cell cycle regulators, including cyclin B1, cyclin D3, CDK4, and CDK6, is also increased, despite that they are not the direct targets of these miRNAs [86]. CircUBE2D2 also contributes to cell cycle progression via sponging miR-512-3p to upregulate the expression of cell division cycle-associated 3 (CDCA3) [87]. Similarly, hsa_circ_0136666 (circPRKDC) stimulates the cell cycle by increasing the expression of CDK6. Without the suppression of miR-1299 that is sponged by circPRKDC, CDK6 abundance can flourish [88]. Using the same mechanism, hsa_circ_0004676 (circDHTKD1) sequesters miR-377-3p, which targets *E2F6* mRNA, thus allowing E2F6 to be translated and further binds to partner of NOB1 homolog (PNO1) promotor to stimulate its expression. PNO1 then induces p53 ubiquitination, impairing its inhibitory effects on the cell cycle [90]. CircUBR1 is overexpressed in breast cancer, and it sponges miR-1299 that targets *CCND1* mRNA. MiR-1299 is subsequently downregulated, while cyclin D1 is upregulated in the presence of circUBR1 [91]. Lastly, hsa_circ_0008039 (circPRKAR1B) supports E2F3 expression via suppressing its inhibitor, miR-432-5p [92].

There are the other circRNAs that mediate the cell cycle through downstream indirect effectors. To illustrate, the high abundance of hsa_circ_0000515 (circRPPH1) decreases the expression of p27, while increases the expression of cyclin D1, and CDK4. Mechanistically, circRPPH1 sponges miR-296-5p, abating the repressive effect on C-X-C motif chemokine ligand 10 (CXCL10) which positively affects the abovementioned cell cycle regulators [93]. CircPLK1 competes with miR-4500 and allows insulin-like growth factor (IGF1) mRNA, a target of miR-4500, to be translated. IGF1 later stimulates the upregulation of CDK4 and CDK6, supporting cell cycle activity [98].

Hsa_circ_0001785 (circELP3) decreases PCNA expression and intervenes in cell cycle progression by targeting miR-942. Subsequently, SOCS3 is overexpressed, inhibiting the JAK/STAT signaling pathway [99,205]. Surprisingly, circFBXW7 utilizes both miRNA sponging and protein translation mechanisms to inhibit breast cancer. It was shown that the protein encoded from circFBXW7 called FBXW7-185aa can bind to USP28 and suppresses c-Myc activity. Moreover, circFBXW7 itself also sponges miR-197-3p to upregulate the expression of its parental gene, F-box/WD repeat-containing protein 7 (FBXW7), which in turn synergizes the inhibitory effect in repressing c-Myc [100]. Another cell cycle-suppressive circRNA, circSETD2, lowers the expression of miR-155-5p by absorbing. This interaction relieves miR-155-5p target, signal peptide CUB domain and EGF like domain containing 2 (SCUBE2), from suppression. SCUBE2 then exerts its inhibitory function on CDK4 and cyclin D1, forestalling the G0/G1 to S phase transition [101].

### 5.3. Cervical Cancer

Most of the circRNAs reported in cervical cancer progression are cell cycle supporters such as circZFR, which upregulates many cell cycle regulators via binding to single-stranded DNA-binding protein 1 (SSBP1). This interaction results in the association of cyclin E1–CDK2 complex, which further phosphorylates Rb, releasing E2F1 and CDK2 expression, respectively [102]. Another example, hsa_circ_0000326 (circTCONS_l2_00004572), which sponges miR-338-3p to upregulate CDK4. Moreover, cyclin D1 expression is increased, whereas p21 and p27 are decreased [105]. Similarly, miR-1296 directly targets *CDK2* mRNA to promote its degradation unless hsa_circ_0000520 (circRPPH1) abolishes the activity by sponging miR-1296. In addition to the upregulation of CDK2 mediated by circRPPH1, the expression of cyclin D1 is also increased, while that of p21 and p27 is decreased [94]. Found abundantly expressed in cervical cancer cell lines, hsa_circ_ 0000263 (circTCONS_00017720) suppresses p53 activity by sequestrating miR-150-5p. MDM4, a negative regulator of p53, is a target of miR-150-5p. Therefore, circTCONS_00017720-mediated miR-150-5p depletion leads to an increase in MDM4 and a decrease in p53, respectively [106]. Hsa_circ_0009035 (circRACGAP1) is also overexpressed in cervical cancer, and it aggravates cell cycle progression by sponging miR-889-3p. Therefore, homeobox B7 (HOXB7), a target of miR-889-3p, can now escape from miRNA-mediated degradation and stimulate PCNA expression [107]. Additionally, hsa_circ_0003221 (circPTK2) increases cytoplasmic polyadenylation element binding protein 4 (CPEB4) by sponging to miR-758-3p, leading to the upregulation of PCNA and cyclin D1 [108]. CircESRP1 supports cyclin D1 expression via suppressing the inhibitory effect of miR-634 and miR-142-3p to increase tumor protein D52 (TPD52) and ADP-ribosylation factor-like protein (ARL2), respectively. The overexpression of TPD52 or ARL2 leads to the upregulation of cyclin D1 and PCNA in the latter [109,110]. As well as hsa_circ_0000285 (circHIPK3) and circASAP1, which sponge miR-197-3p and miR-338-3p, respectively. MiR-197-3p represses ELK1 mRNA, while miR-338-3p interferes with RPP25 mRNA translation. The increased expression of PCNA and cyclin D1 can be observed when overexpressing ELK1 or RPP25 [111,112].

### 5.4. Colon Cancer

Hsa_circ_0008231 (circNBPF11) enhances colon cancer proliferation via PI3K/Akt signaling by endogenously competing with miR-338-3p. Without miR-338-3p-mediated repression, ETS proto-oncogene 1 (ETS1) expression is then increased and triggers phosphorylation of the upstream players of PI3K/Akt signaling, such as PI3K and Akt. Therefore, the upregulation of cyclin D1 is then followed [114]. Another study reported that circCTIC1 employs a different approach to modulate tumor-initiating cells in colon cancer. CircCTIC1 interacts with bromodomain PHD finger transcription factor (BPTF), one of the crucial partners of nuclear remodeling factor (NURF) complex, to mediate the localization into the nucleus. NURF complex binds to c-Myc promotor and thus increases c-Myc expression, promoting self-renewal capacity [115]. In the other way, circCSPP1 positively regulates Rho-associated coiled-coil containing protein kinase 1 (ROCK1) abundance through miR-431 inhibition. ROCK1 further promotes cyclin D1 and CDK4 expression, which, in turn, phosphorylate Rb to allow the G0/G1-S phase transition [116].

### 5.5. Colorectal Cancer

Utilizing Wnt/β-catenin signaling, hsa_circ_0005615 (circNFATC3) reinforces colorectal cancer cell behavior through increasing the expression of tankyrase (TNKS) by sponging miR-149-5p [117]. In the absence of Wnt, β-catenin is captured by the protein complex containing AXIN, adenomatous polyposis coli (APC), casein kinase 1 (CK1), and glycogen synthase kinase 3 (GSK-3), preventing β-catenin from functioning [206]. However, TNKS abolishes the suppressive effects of the complex by inhibiting AXIN to release β-catenin. Consequently, cyclin D1 expression is then mediated [117]. Unlike circNFATC3, circMETTL9 directly supports CDK6 expression through the inhibition of miR-551b-5p [118]. Similarly, the suppressive effect of miR-377 on *E2F3* mRNA can be reversed by circPRMT5, that acts as a competing endogenous RNA [119]. Another example, hsa_circ_0007142 (circDOCK1) also sponges miR-122-5p, allowing its target CDC25A to express [120].

Nonetheless, there are circRNAs that can promote the cell cycle through indirect effectors. To illustrate, circCTNNA1 absorbs miR-149-5p to upregulate forkhead box protein M1 (FOXM1), a transcriptional activator of cyclin B1 and cyclin D1. This results in the rise of cyclin B1 and cyclin D1, along with the significant downregulation of p21 and p27 [121]. CircPRKDC and hsa_circ_0000512 (circRPPH1) promote cyclin D1 expression via different miRNAs and their downstream targets. CircPRKDC sponges miR-198 to increase discoidin domain receptor tyrosine kinase 1 (DDR1), whereas circRPPH1 binds to miR-296-5p to upregulate RUN family transcription factor 1 (RUNX1). Both DDR1 and RUNX1 further positively induce cyclin D1 levels [89,95]. Employing a similar approach, circZFR represses miR-147a to abrogate the inhibitory effect on CDK2-associated cullin domain 1 (CACUL1). Consequently, the increased expression of CDK2, CDK4, CDK6, and cyclin D1 is then conveyed by CACUL [103].

### 5.6. Endometrial Cancer

Hsa_circ_0002577 (circWDR26) was found to be upregulated in endometrial cancer tissue and cell lines. Moreover, circWDR26 modulates cell cycle progression by controlling the Wnt/β-catenin signaling pathway. As miR-197 interferes with the translation of catenin delta 1 (CTNND1), circWDR26 absorbs miR-197 to restore CTNND1 expression. CTNND1 thereby activates Wnt/β-catenin signaling, resulting in a surge of β-catenin, cyclin D1, and c-Myc [122]. In contrast to circWDR26, hsa_circ_0001610 (circTNFRSF21) uses a straightforward approach to promote cyclin B1 by repressing its suppressor, miR-139-5p [123].

### 5.7. Esophageal Cancer

Normally found upregulated, circFIG4 promotes cell cycle activity through sponging miR-493-5p, releasing E2F3 from being repressed [124]. Interestingly, circFNDC3B and hsa_circ_00014879 (circDCAF8) positively influence the same downstream target, CDC25A. CircFNDC3B mediates the action through inhibiting miR-214-3p, whereas circDCAF8 suppresses miR-519-3p [125,126]. Hsa_circ_0003340 (circOGDH) was reported to absorb miR-940 and modulate its downstream target expression, protein kinase AMP-activated catalytic subunit alpha 1 (PRKAA1). PRKAA1 can induce cyclin D1 and PCNA expression to positively regulate the cell cycle [127]. Similarly, hsa_circ_0120816 (circGFPT1) participates in cell cycle progression by sequestering miR-1305, which inhibits thioredoxin reductase 1 (TXNRD1) expression. Consequently, the upregulation of TXNRD1 is then mediated, followed by the increase in cyclin B1 induced by TXNRD1 [128].

### 5.8. Gastric Cancer

CircPGPEP1 was revealed to stimulate the G0/G1 to S phase transition as it targets miR-1297 to directly increase E2F3 abundance [129]. Likewise, hsa_circ_104433 competes with miR-497-5p, leading to the significant expression of its target, CDC25A. Interestingly, cyclin B1 was also found to be positively correlated to hsa_circ_104433 level [130]. Unlike the previous example, circRNF111 sponges miR-876-3p to restore Krüppel-like Factor 12 (KLF12). KLF12 subsequently mediates the upregulation of cyclin D1, thus supporting cell cycle [131].

On the other hand, circRNAs that act against cell cycle are, indeed, found to be downregulated in gastric cancer tissue and cell lines. To begin with, hsa_circ_0006470 (circMFN2) promotes p53 inducible protein 11 (TP53I11) by sponging miR-1234. The upregulation of TP53I11 prevents Akt phosphorylation and leads to the inhibition of the PI3K/Akt signaling pathway. Furthermore, TP53I11 also stimulates FOXO1 synthesis to suppress the activity of CDK2 [133]. As with circEVI5, it sequesters miR-4793-3p to relieve FOXO1 expression, followed by the encoding of p21 and p27 [134].

### 5.9. Glioma

Hsa_circ_0001495 (circCCNB1) can be induced by a transcription factor, eukaryotic translation initiation factor 4A3 (EIF4A3). CircCCNB1 employs multiple mechanisms for modulating glioma aggressiveness. It recruits HuR, an RNA-binding protein that functions as a stabilizer of *CCND1* mRNA. Moreover, circCCNB1 simultaneously sponges miR-516b-5p to prevent miRNA-mediated degradation of *CCND1*, resulting in an increased cyclin D1 protein level [135]. Utilizing only miRNA sponge capability, hsa_circ_0001982 (circRNF111) sequesters miR-1205, which is an inhibitor of *E2F1* mRNA. Due to the lack of miR-1205 presence, E2F1 is freed from repression and participates in the S phase transition [132]. Nonetheless, hsa_circ_0012129 (circB4GALT2) and hsa_circ_0030018 (circPOSTN) can also promote the progression of glioma without the direct control of cell cycle-related proteins. CircB4GALT2 upregulates TGIF2 expression through sponging miR-761. Subsequently, TGIF2 boosts the abundance of CDK2 and CDK4 to proceed to the S phase [136]. In a similar manner, circPOSTN absorbs miR-1297 to increase RAB21 level, which then facilitates the elevation of cyclin D1 and PCNA [137].

DNA damage in gliomas can induce circCDR1as, which serves as a protein sponge to attract p53. The interaction prevents p53 from establishing a complex with MDM2, its negative regulator [138]. MDM2 blocks transcriptional activation and subjects p53 for degradation mediated by ubiquitination [207]. Consequently, p53 and its downstream effector p21 are then increased, forestalling the cell cycle of glioma cells [138].

### 5.10. Hepatocellular Carcinoma

The majority of circRNA involving cell cycle activity in hepatocellular carcinoma is upregulated in both tumor tissue and cell lines. In addition, most of them are classified as cell cycle supporters. To begin with, hsa_circ_0036412 (circETFA) manipulates the Hedgehog signaling network through binding to ELAV like RNA-binding protein 1 (ELAVL1), an RNA-binding protein. ELAVL1 stabilizes *GLI2* mRNA and increases protein levels [139]. GLI2 is an essential regulator in the Hedgehog signaling pathway. A previous study showed that downregulation of GLI2 negatively affects hepatocellular carcinoma proliferation due to the increase in p21 and p27 expression [208]. High GLI2 abundance mediated by circETFA then stimulates cyclin D1. In addition, circETFA utilizes miRNA sponge capability to synergistically enhance GLI2 production by sponging its suppressor, miR-579-3p [139].

Hsa_circ_0091581 (circGPC3) efficiently promotes hepatocellular carcinoma proliferation by establishing a positive feedback loop. To illustrate, miR-526b, which is a repressor of *c-Myc* mRNA, is sponged by circGPC3, thus upregulating c-Myc translation. The increase in c-Myc further stimulates circGPC3 production and cell cycle progression [140]. Hsa_circ_0000519 (circRPPH1) facilitates the G0/G1 to S phase transition through sponging miR-1296. Identified as one of the targets of miR-1296, E2F7 becomes upregulated when overexpressed with cirRPPH1. Interestingly, cyclin D1 and cyclin E1 were also found to be elevated, even though they are not direct targets to miR-1296 [96]. Another example, circROBO1 absorbs miR-130a-5p, which inhibits *CCNT2* mRNA translation. This results in the reduction of miR-130a-5p, followed by the elevation of cyclin T2 level and allows the cell cycle to proceed to the M phase [142]. CircMMP9 acts via the miR-149/CCND2 axis to support cell cycle. Upon the downregulation of miR-149 by circMMP9, cyclin D2 expression is then increased [143]. Hsa_circ_101555 elevates CDCA3 expression by sponging its inhibitor, miR-145-5p. In the absence of miR-145-5p, CDCA3 abundance is thus more evident [144]. CircZNF83 also participates in cell proliferation via modulating indirect downstream targets. MiR-324-5p, a suppressor of CDK16 and a target of circZNF83, is then downregulated, allowing the expression of CDK16 to thrive. Moreover, circZNF83 relies on the JAK/STAT pathway to simultaneously aggravate cancer behavior, as the phosphorylated JAK2 and STAT3 were found to be increased. Nonetheless, the expression of the other cell cycle regulators has not been examined [145]. Lastly, circSLC7A11 endogenously silences miR-330-3p, which targets *CDK1* mRNA, resulting in the downregulation of miR-330-3p and the upregulation of cyclin D1 expression, respectively. In addition, an increase in cyclin B1 level can be observed in circSLC7A11-enriched condition [146].

As previously mentioned, circGPC3 greatly supports cell cycle activity. A recent study showed that the other circular transcript of circGPC3, hsa_circ_0091579, can also contribute to cell cycle progression. Mechanistically, hsa_circ_0091579 sponges miR-1287 to inhibit miR-1287-mediated mRNA degradation on pyruvate dehydrogenase kinase 2 (PDK2) mRNA. An elevated PDK2 level positively influences cyclin D1 expression [141]. Similarly, hsa_circ_0005397 (circRHOT1), which is originally overexpressed in hepatocellular carcinoma, also sequesters miR-326 to upregulate PDK2. Indeed, an increase in cyclin D1 abundance can be detected, along with a decrease in p21 expression [147]. Hsa_circ_0000517, the other circular transcript of circRPPH1, promotes the transition of the cell cycle to the S phase by sponging miR-326. After circRNA-mediated sequestration, miR-326 level is then declined, which leads to the upregulation of its target, SMAD6. Cyclin D1 expression is subsequently upregulated under the influence of SMAD6 [97].

CircVAMP3 participates in anti-cell cycle activity as it features protein binding capability and acts against cell proliferation. Mechanistically, circVAMP3 directly binds to cell cycle-associated protein 1 (CAPRIN1) to block the interaction with G3BP stress granule assembly factor 1 (G3BP1). Without the complex resulting from the association between CAPRIN1 and G3PB1, the activation of c-Myc is restrained [148]. Another example, circLARP4, absorbs miR-761 to restore RUNX3. Thus, RUNX3 expression is elevated during miR-761 depletion and then positively influences p53 and p21 expression, inhibiting the cell cycle [149]. As well as hsa_circ_ 0000204 (circWDR37), which acts as cell cycle-suppressive circRNA. Through sponging miR-191, circWDR37 can upregulate KLF6 expression, which further inhibits cyclin D1, cyclin D2, and c-Myc [150].

### 5.11. Leukemia

As previously described, some circRNAs can be synthesized from fused genes in cancer, such as F-circBA1, which resulted from the fusion of *BCR* and *ABL* gene. A study indicated the cell cycle supportive role of F-circBA1 as it aggravates the transition of the G2 to M phase in chronic myeloid leukemia. F-circBA1 binds to miR-148b-3p which suppresses *CDC25B* mRNA translation, thus upregulating CDC25B abundance [28]. Hsa_circ_0005774 (circCDK1) sponges miR-192-5p to increase the expression of uncoordinated 51-like kinase 1 (ULK1). The report showed that ULK1 is a positive regulator of cyclin D1 and PCNA because these proteins are increased in the presence of ULK1 [151]. As well as hsa_circ_0094100 (circSFMBT2), it increases the upregulation of cyclin D1 and PCNA through the miR-217/ATP1B1 axis. The decline of miR-217 mediated by circSFMBT2 results in the elevation of ATPase Na^+^/K^+^-transporting subunit beta 1 (ATP1B1), which further stimulates cyclin D1 and PCNA [152]. In addition, circNFIX was found to upregulate the expression of cyclin D1. By sponging miR-876-3p, circNFIX can increase the level of tripartite motif containing 31 (TRIM31), which then activates the synthesis of cyclin D1 [153].

### 5.12. Lung Cancer

EGFR stimulation can lead to the synthesis of hsa_circ_0000190 (circCNIH4), an upregulated circRNA in non-small-cell lung cancer (NSCLC). CircCNIH4 inhibits miR-142-5p to further restore CDK6 expression. In addition, circCNIH4 also takes advantage of the MAPK signaling pathway by increasing the phosphorylation of MEK1/2 and ERK1/2. Therefore, the downstream targets of the pathway, such as CDK1, CDK4, and phosphorylated Rb, are upregulated [154]. Another circRNA that modulates cell signaling to facilitate cell cycle progression is hsa_circ_0014235 (circS100A2). MiR-146b-5p naturally interferes with the translation of Yes-associated protein (*YAP*) mRNA, which is classified as one of the key regulators in Hippo signaling [155]. Previous studies reported that YAP is involved in the activation of E2F, CDK2, CDK4, and CDK6 [209]. The increase in YAP mediated by the circS100A2/miR-146b-5p axis thus facilitates the production of CDK4 [155]. Moreover, circS100A2 can also utilize the miRNA sponge function to bind miR-520a-5p, which is a suppressor of CDK4 [156]. Taken together, the evidence indicates that circS100A2 can effectively upregulate CDK4 expression.

To upregulate cyclin E1, circDENND2A and hsa_circ_0062389 (circPI4KA) sponge miR-34a and miR-103a-3p, respectively. Coincidentally, miR-34a and miR-103a-3p repress *CCNE1* mRNA translation. Furthermore, circDENND2A and circPI4KA are naturally overexpressed in NSCLC, thus leading to the efficient upregulation of cyclin E1 [157,158]. Hsa_circ_0012384 (circCMPK1) sponges miR-302e to relieve cyclin D1 from suppression [159]. As well as circABCB10, it sequesters miR-584-5p, which inhibits the translation of E2F5 mRNA. As a result, E2F5 is indeed upregulated in the presence of circABCB10 [160]. The same mechanism can be found in hsa_circ_0001421 (circSEC31A). *CDCA3* mRNA is repressed by miR-4677-3p, but circSEC31A abolishes this effect by sponging miR-4677-3p and elevating CDCA3, respectively [161]. Another example, circSATB2, is overexpressed in NSCLC, and it sponges miR-33a-5p to support cancer proliferation. Without miRNA-mediated inhibition, E2F7, which is one of the targets of miR-33a-5p, can thrive [162]. Lastly, circPITX1 endogenously competes with miR-1248 to upregulate cyclin D2 expression, while hsa_circ_0006692 (circTCONS_00025531) binds to miR-205-5p to increase CDK19, along with some regulators that are non-miRNA targets, such as cyclin D1 and PCNA [163,164].

Many circRNAs also modulate lung cancer through the production of indirect effectors. For example, circTP63 sponges miR-873-3p to stimulate the expression of FOXM1, thus further activating the activity and expression of cyclin B1 [165]. Another example, circFOXM1, upregulates cyclin D1 and cyclin E1 levels through the miR-614/FAM83D axis. Upon suppression by circFOXM1, miR-614 is then declined, which facilitates the expression of FAM83D, a positive regulator of cyclin D1 and cyclin E1 [166]. A similar path of action can also be found in hsa_circ_0046263 (circP4HB), which binds to miR-940 to prevent repressive function in NOVA alternative splicing regulator 2 (NOVA2). Next, NOVA2 triggers the abundance of cyclin D1 and PCNA [167]. As well as hsa_circ_0072088 (circZFR), which facilitates the expression of NOVA2 through sponging miR-377-5p. An increase in cyclin D1 and PCNA is then followed [104].

Hsa_circ_0065214 (circSCAP) recruits splicing factor 3a subunit 3 (SF3A3) and induces ubiquitin-mediated degradation via proteasome. The decline in SF3A3 allows its suppressed target, MDM4 exon 6 skipping transcript (MDM4-S), to upregulate and eventually activate p53. In addition, the expression of CDK2, CDK4, CDK6, and cyclin D1 is depressed under circSCAP influence [168]. Likewise, circNOL10 directly binds to sex comb on midleg-like 1 (SCML1), a transcription factor, to prevent ubiquitination and facilitate transcription. Consequently, members of humanin (HN) polypeptide family, HN2 and HN8, are then transcribed and translated. HN2 and HN8 trigger the phosphorylation of checkpoint CHEK2, CDC25A, and E2F1 and activate p53. Moreover, it also decreases cyclin D1, CDK2, and phosphorylated Rb, thus impeding the cell cycle [169]. Employing a less complicated approach, circFBXO7 sequesters miR-296-3p, leading to the expression of KLF15. Acting as a transcription factor, KLF15 translocates to the nucleus and binds to p21 promotor to initiate its transcription [170]. Similarly, circKIF20B sponges miR-615-3p to upregulate myocyte enhancer factor 2A (MEF2A), which later suppresses CDK4 expression [210].

### 5.13. Melanoma

Hsa_circ_0082835 (circEZH2) sponges miR-429, which inhibits its parental gene expression, EZH2. With a lower miR-429 level, EZH2 is relieved from repression and further activates Wnt5a and β-catenin, which are important components in the Wnt/β-catenin signaling network. Indeed, downstream cell cycle regulators, including cyclin D1 and cyclin E1, are then upregulated [171]. Similarly, hsa_circ_0062270 (circCDC45) mediates melanoma phenotype by modulating its parental gene product, CDC45, and functions as a protein sponge as well. EIF4A3, an RNA-binding protein, is recruited to bind to circCDC45 and assist CDC45 mRNA stabilization. This results in an increase in CDC45 protein levels and promotes cell cycle [172].

### 5.14. Multiple Myeloma

CircPSAP promotes the cell cycle through the miR-331-3p/histone deacetylase 4 (HDAC4) axis. The suppression of miR-331-3p mediated by circPSAP leads to the upregulation of HDAC4, which further stimulates cyclin D1 expression to drive the transition to the S phase [173].

As it is downregulated in multiple myeloma, circMYBL2 exerts its anti-cancer effect by facilitating the complex formation of cyclin F and its parental gene product, MYBL2. This prevents the phosphorylation of MYBL2, a transcriptional regulator of several oncogenes, thus inactivating MYBL2 and impeding cell cycle progression [174].

### 5.15. Oral Squamous Cell Carcinoma

Hsa_circ_0000745 (circSPECC1) combines both protein binding and miRNA sponge function to upregulate cyclin D1. To illustrate, circSPECC1 recruits HuR to assist *CCND1* mRNA stabilization. Moreover, it absorbs miR-488, which suppresses CCND1 translation. Therefore, circSPECC1 can efficiently promote cyclin D1 abundance [175]. Another example is circOSBPL10, a downregulated circRNA that also acts against cell cycle by downregulating miR-299-3p. Without the repression from miR-299-3p, its target, CDK6, can be overexpressed. Due to its downregulated status in oral squamous cell carcinoma tissue and cell lines, cell cycle supportive function of circOSBPL10 is usually disabled [176].

Utilizing the same approach as circSPECC1, circYAP also features protein binding capability to ameliorate oral squamous cell carcinoma aggression. CDK4 is recruited to the binding site located within circYAP. This interaction prevents complex formation between CDK4 and cyclin D1 and impairs nucleus translocation. Thus, cyclin D1 expression in the nucleus is diminished, including other regulators such as PCNA, c-Myc, and Rb [177].

### 5.16. Osteosarcoma

Through the circARF3/miR-1299/CDK6 axis, the naturally overexpressed circARF3 can readily sponge miR-1299 in osteosarcoma cell lines. The downregulation of miR-1299 results in the elevation of CDK6, thus promoting the G0/G1 to S phase transition [178]. Hsa_circ_0008934 (circASAP1) is also known to support E2F3 expression by sponging its suppressor, miR-145-5p. Consequently, E2F3 abundance becomes more pronounced in the absence of miR-145-5p [113]. Similarly, hsa_circ_0000073 (circOMA1) mediates cyclin E2 and MDM2 expression via miR-1252-5p. Both cyclin E2 and MDM2 have been proven to be targets of miR-1252-5p. Therefore, circOMA1-mediated miR-1252-5p repression leads to the upregulation of cyclin E2 and MDM2 [179]. Hsa_circ_0084582 (circCHD7) modulates the cell cycle through sponging miR-485-5p. Identified as a target of miR-485-5p, jagged 1 (JAG1) can be upregulated upon the suppression of miR-485-5p by circCHD7. JAG1 next stimulates c-Myc expression and aggravates osteosarcoma phenotype [180]. Another example, circSAMD4A, was found to be significantly overexpressed in doxorubicin-resistant osteosarcoma tissue and cell lines. It competes with miR-218-5p that targets KLF8, thus leading to the reduction in miR-218-5p and the promotion of KFL8, respectively. Under KLF8 influence, cyclin D1 is then upregulated, whereas p21 is downregulated [181].

### 5.17. Ovarian Cancer

Using a similar mode of action to circCNNB1 in glioma, circE2F2 serves as a protein sponge to recruit HuR to the binding site. HuR subsequently promotes E2F2 mRNA stabilization and therefore increases E2F2 protein level [182]. The other circRNA that supports the cell cycle in ovarian cancer is hsa_circ_0000714 (circAARS). The study reported that circAARS was found to be upregulated in paclitaxel-resistant ovarian tissue and cell lines. Through absorbing miR-370-3p, circAARS can mediate RAB17 expression, which further stimulates CDK6 and Rb, aggravating cancer behavior [183].

### 5.18. Pancreatic Ductal Adenocarcinoma

CircNEIL3 exerts its cell cycle supportive role by sponging miR-432-5p which, in turn, targets adenosine deaminase acting on RNA 1 (ADAR1). ADAR1 induces adenosine-to-inosine (A-to-I) editing of GLI family zinc finger 1 (GLI1) and performs a positive feedback loop to stimulate the production of circNEIL3 [184]. As with GLI2, which was previously mentioned, GLI1 is also considered a downstream important key in the Hedgehog pathway [211]. The evidence reported that GLI1 depletion led to the downregulation of many cell cycle regulators [212]. Therefore, the upregulation of ADAR1-induced GLI1 results in a surge of cyclin D1, cyclin E1, CDK2, CDK4, and CDK6 [184].

CircPVRL3 represses several cell cycle regulators by relying on the transactivation between the JAK/STAT and PI3K/Akt pathways [185]. To illustrate, the expression of Akt can be abolished by silencing STAT3 or JAK2 [213]. Another study showed that trans-signaling of the JAK/STAT pathway mediated by IL-6 also activates both the PI3K/AKT and MAPK networks, indicating the importance of JAK/STAT signaling to the PI3K/Akt pathway [214]. CircPVRL3 absorbs miR-194-5p, which is a repressor of suppressor of cytokine signaling 2 (SOCS2), an inhibitor of JAK/STAT. The high expression of SOCS2 is then mediated to inactivate the pathway, negatively affecting PI3K/Akt signaling in the process. Thus, many regulators driving the cell cycle, such as cyclin D1, cyclin E1, CDK2, and CDK4, are declined, whereas p21 is increased instead [185].

### 5.19. Prostate Cancer

Few studies were conducted to examine the role of circRNA and cell cycle-related proteins. For instance, hsa_circ_0057558 (circSLC39A10) was demonstrated to boost c-Myc, cyclin B1, and cyclin D1 expression via inhibiting miR-206 activity. Ubiquitin specific peptidase (USP33) is identified as a target of miR-206, and its function is to deubiquitinate c-Myc, thus allowing c-Myc to escape degradation and to upregulate the abovementioned proteins [186]. However, circHMGCS1 employs different mechanisms for enhancing cell cycle. By sponging miR-205-5p, its downstream target, Erb-B2 receptor tyrosine kinase 3 (ErBB3), is then upregulated. ErBB3 promotes phosphorylation of Akt and PI3K to activate the PI3K/Akt signaling pathway, resulting in an increase in cyclin D1 and CDK4 [187]. Directly bound to *CCND1* mRNA, miR-195 can impede its translation into cyclin D1. However, miR-195 is usually repressed by circDPP4 in prostate cancer cell lines, thus facilitating cyclin D1 expression [188]. Lastly, circDPP4 functions as a sponge to attract miR-195, a repressor of *CCND1*. The reduction in miR-195 level is then mediated, followed by the elevation of cyclin D1, respectively [188].

### 5.20. Thyroid Cancer

Functioning as a protein recruiter, circEIF3I attracts AU-rich element RNA-binding factor 1 (AUF1) to the binding site. AUF1 is then occupied and cannot be used to trigger *CCND1* mRNA degradation. Subsequently, cyclin D1 is significantly increased [189]. CircPRKCI and hsa_circ_0000644 (circKIAA1199) were identified to share the same target E2F3, yet they mediate through different miRNAs. CircPRKCI binds to miR-335 while circKIAA1199 sponges miR-1205 [190,191].

CircHACE1 exhibits an anti-cell cycle in thyroid cancer cells through the miR-346/TFC2L1 axis. The overexpression of circHACE1 leads to a reduction in miR-346 levels and an increase in TFC2L1, respectively. TFC2L1 then downregulated cyclin B1 and PCNA expression, therefore impeding the cell cycle [192].

### 5.21. Other Cancers

In laryngeal carcinoma, a naturally overexpressed circMYLK aggravates cancer cell proliferation by sponging miR-195. Consequently, *CCND1* mRNA, a target of miR-195, is free from its repressor and can be translated into cyclin D1 [78]. As found upregulated in nasopharyngeal carcinoma, circCAMSAP1 manages to reinforce the cancer phenotype through bizarre function. Rather than recruiting to miRNA or protein, circCAMSAP1 directly binds and manipulates serpin family H member 1 (SERPINH1) mRNA, enhancing stabilization. High expression of SERPINH1 prevents the ubiquitination and degradation of c-Myc. Moreover, the positive feedback loop can also be stimulated by the complex formation of c-Myc and splicing factor 10 (SRSF10), resulting in the upregulation of circCAMSAP1 [193]. CircFAM114A2 acts against cell cycle activity in urothelial carcinoma by sponging miR-222-3p and miR-146a-5p. Both miRNAs target negative cell cycle regulators. MiR-222-3p suppresses p27 translation, whereas miR-146a-5p targets p21. Thus, the overexpression of circFAM114A2 leads to the expression of p27 and p21 [194].

## 6. Conclusions and Future Perspectives

Cancer is a global health issue and is considered life threatening. An aberrant expression of cell cycle regulators combined with a modulation of cellular signaling is one of the key driving factors that aggravates cancer behavior. Emerging research indicated that circRNAs might play an important role in regulating cancer aggressiveness since they are most likely to be dysregulated in cancer tissues and cell lines. CircRNAs can serve as either cell cycle supporters or cell cycle inhibitors, depending on the expression status, miRNA targets, mRNA targets, or protein targets. Most circRNAs have been reported to serve as miRNA sponges. The mounting evidence suggests that they might also hold other molecular mechanisms such as protein sponge, protein translation, mRNA sponge, and guiding A-to-I editing [215]. However, many studies have only identified a brief correlation between circRNAs and cell cycle activity, without examining whether circRNAs indeed cause the altered expression of cell cycle regulators.

Due to the advancement of bioinformatics tools and high-throughput RNA sequencing that have been rapidly developing, the identification of novel modes of circRNA-mediated gene regulation has thus gained more attention. A recent study demonstrated that circRNA–miRNA interactions can be predicted using a combination of deep learning and algorithms called KGDCMI. RNA characteristics in the form of digitalized data can be retrieved from algorithms before constructing a possible interaction network. Lastly, the calculation of the final predicted score will be made. The prediction accuracy was considerably high compared to that of the existing model, indicating the effectiveness of KGDCMI [216]. Other than predicting circRNA–miRNA interactions, many bioinformatics tools have also been recently developed for the prediction of circRNA–protein interactions in recent years. Recently, circRNA regulator identification tool (CRIT) has utilized multiple datasets such as gene alteration, clinical significance, gene ontology (GO) similarity, and transcriptome data collected from twelve cancer types. All the data are subjected to being characterized and clustered using non-negative matrix factorization (NMF), resulting in 73 predicted candidates and 17 known candidates [217]. Therefore, CRIT might be another efficient tool for predicting circRNA–protein interactions, as it revealed both novel and known regulators. These recent bioinformatics tools are undoubtedly useful for the future discovery of circRNA modulating cell cycle regulation in cancers.

In addition to the growing trend in establishing prediction tools, many molecular techniques have also been developed, especially for the study of circRNA–protein interaction. Employing RNA immunoprecipitation (RIP) might be effective in revealing RBPs, but it would require a suitable antibody. Thus, electrophoretic mobility shift assays (EMSAs) are more appropriate for determining the interaction between circRNAs and candidate proteins. Basically, both circRNA and RBPs are incubated in the mixture before being subjected to electrophoresis. Free circRNA tends to migrate faster than circRNA-bearing RBPs [15]. Nonetheless, disadvantages persist because binding circRNAs to RBPs necessitates a stable condition during gel run, and large circRNAs may not be suitable for this approach [218]. In addition, these interactions may not be physiologically present in the cells. Although these methods have their limitations, they may help us learn more about how circRNAs and their RBPs play a role in cell cycle dysregulation. Moreover, it could reveal the possibility of discovering biomarkers and therapeutic targets.

Given that circRNAs are abnormally expressed in each organ or cancer tissue, measuring their quantity would be a promising diagnosis. Reports suggest that the aggressiveness of the tumor influences the expression of some circRNAs. However, there are still some limitations, as it is common to find circRNAs in chunks and in extremely low abundance, which can complicate the process of identifying the specific one. Moreover, investigating the significance of circRNAs from biological samples, especially blood and plasma, is also considered a long procedure and may lead to incomplete identification [219,220]. Therefore, huge developments and research are required before utilizing circRNAs as a biomarker for disease diagnosis in clinical practice.

## Figures and Tables

**Table 1:** CircRNAs and their functions in modulating cell cycle regulators throughout different cancers.

CircRNAs	Function	Target miRNA	Target mRNA	Target Protein	Molecular Mechanism	Disease	Role and Expression Status	Ref.
Hsa_circ_0071196 (circLRBA)	miRNA sponge	miR-19b-3p	CIT	CIT	Inhibit miR-19b-3p to upregulate CIT, a positive regulator of the MAPK pathway, leading to an increase in CDK1 and cyclin D1 expression	Bladder cancer	Oncogenic & Upregulated	[73]
Hsa_circ_0132246 (circRIMS1)	miRNA sponge	miR-433-3p	CCAR1	CCAR1	Inhibit miR-433-3p to upregulate CCAR1, a coactivator of β-catenin, leading to an increase in c-Myc expression	Bladder cancer	Oncogenic & Upregulated	[74]
Hsa_circ_0058063 (circATIC)	miRNA sponge	miR-1485-5p	CDK6	CDK6	Upregulate CDK6 by inhibiting its repressor, miR-1485-5p	Bladder cancer	Oncogenic & Upregulated	[75]
Hsa_circ_0000615 (circZNF609)	miRNA sponge	miR-1200	CDC25B	CDC25B	Upregulate CDC25B by inhibiting its repressor, miR-1200	Bladder cancer	Oncogenic & Upregulated	[76]
CircMYLK	miRNA sponge	miR-34a	CCND3	Cyclin D3	Upregulate CCND3 by inhibiting its repressor, miR-34a	Bladder cancer	Oncogenic & Upregulated	[77]
	miRNA sponge	miR-195	CCND1	Cyclin D1	Upregulate cyclin D1 by inhibiting its repressor, miR-195	Laryngeal carcinoma	Oncogenic & Upregulated	[78]
CircGLIS3	miRNA sponge	miR-1273f	SKP1	SKP1	- Promote SKP1 expression by inhibiting its repressor, miR-1273f - SKP1 positively regulates cyclin D1	Bladder cancer	Oncogenic & Upregulated	[79]
CircBCRC-3	miRNA sponge	miR-182-5p	P27	P27	Upregulate P27 by inhibiting its repressor, miR-182-5p	Bladder cancer	Anti-oncogenic & Downregulated	[80]
CircITCH	miRNA sponge	miR-17	P21	P21	Upregulate P21 by inhibiting its repressor, miR-17	Bladder cancer	Anti-oncogenic & Downregulated	[81]
		miR-24	PTEN	PTEN	Inhibit miR-24 to upregulate PTEN, a negative regulator of the PI3K/Akt pathway			
Hsa_circ_0006117 (circPTPRA)	Protein sponge	-	-	IGF2BP1	Block IGF2BP1 from recognizing m^6^A-modified mRNAs, such as *c-Myc*, and promoting stability and translation	Bladder cancer	Anti-oncogenic & Downregulated	[82]
CircNR3C1	miRNA sponge	miR-27a-3p	CCND1	Cyclin D1	Downregulate cyclin D1 by inhibiting its activator, miR-27a-3p	Bladder cancer	Anti-oncogenic & Downregulated	[83]
Hsa_circ_0091074 (circTCONS_00016926)	miRNA sponge	miR-1297	TAZ	TAZ	Inhibit miR-1297 to upregulate TAZ, a downstream effector of the Hippo pathway, leading to an increase in CDK4, CDK6, and cyclin D1 expression	Breast cancer	Oncogenic & Downregulated	[84]
CircPGR	miRNA sponge	miR-301a-5p	CDK1, CDK6, CHEK2	CDK1, CDK6, CHEK2	Upregulate CDK1, CDK6, and CHEK2 by inhibiting their repressor, miR-301a-5p	Breast cancer	Oncogenic & Upregulated	[85]
CircHMCU	miRNA sponge	Let-7 family	c-Myc, CCND1	c-Myc, Cyclin D1	Upregulate c-Myc and cyclin D1 by inhibiting their repressor, let-7 family	Breast cancer	Oncogenic & Upregulated	[86]
CircUBE2D2	miRNA sponge	miR-512-3p	CDCA3	CDCA3	Upregulate CDCA3 by inhibiting its repressor, miR-512-3p	Breast cancer	Oncogenic & Upregulated	[87]
Hsa_circ_0136666 (circPRKDC)	miRNA sponge	miR-1299	CDK6	CDK6	Upregulate CDK6 by inhibiting its repressor, miR-1299	Breast cancer	Oncogenic & Upregulated	[88]
	miRNA sponge	miR-198	DDR1	DDR1	- Upregulate DDR1 by inhibiting its repressor, miR-198 - DDR1 positively regulates cyclin D1	Colorectal cancer	Oncogenic & Upregulated	[89]
Hsa_circ_0004676 (circDHTKD1)	miRNA sponge	miR-377-3p	E2F6	E2F6	- Upregulate E2F6 by inhibiting its repressor, miR-377-3p - E2F6 stimulates PNO1 to further induces p53 ubiquitination	Breast cancer	Oncogenic & Upregulated	[90]
CircUBR1	miRNA sponge	miR-1299	CCND1	Cyclin D1	Upregulate cyclin D1 by inhibiting its repressor, miR-1299	Breast cancer	Oncogenic & Upregulated	[91]
Hsa_circ_0008039 (circPRKAR1B)	miRNA sponge	miR-432-5p	E2F3	E2F3	Upregulate E2F3 by inhibiting its repressor, miR-432-5p	Breast cancer	Oncogenic & Upregulated	[92]
Hsa_circ_0000515 (circRPPH1)	miRNA sponge	miR-296-5p	CXCL10	CXCL10	- Upregulate CXCL10 by inhibiting its repressor, miR-296-5p - CXCL10 positively regulates CDK4, and cyclin D1 and negatively regulates p27	Breast cancer	Oncogenic & Upregulated	[93]
Hsa_circ_0000520 (circRPPH1)	miRNA sponge	miR-1296	CDK2	CDK2	Upregulate CDK2 by inhibiting its repressor, miR-1296	Cervical cancer	Oncogenic & Upregulated	[94]
Hsa_circ_0000512 (circRPPH1)	miRNA sponge	miR-296-5p	RUNX1	RUNX1	- Upregulate RUNX1 by inhibiting its repressor, miR-296-5p - RUNX1 positively regulates cyclin D1	Colorectal cancer	Oncogenic & Upregulated	[95]
Hsa_circ_0000519 (circRPPH1)	miRNA sponge	miR-1296	E2F7	E2F7	Upregulate E2F7 by inhibiting its repressor, miR-1296	Hepatocellular carcinoma	Oncogenic & Upregulated	[96]
Hsa_circ_0000517 (circRPPH1)	miRNA sponge	miR-326	SMAD6	SMAD6	- Upregulate SMAD6 by inhibiting its repressor, miR-326 - SMAD6 positively regulates cyclin D1	Hepatocellular carcinoma	Oncogenic & Upregulated	[97]
CircPLK1	miRNA sponge	miR-4500	IGF1	IGF1	- Upregulate IGF1 by inhibiting its repressor, miR-4500 - IGF1 positively regulates CDK4, and CDK6	Breast cancer	Oncogenic & Upregulated	[98]
Hsa_circ_0001785 (circELP3)	miRNA sponge	miR-942	SOCS3	SOCS3	Inhibit miR-942 to upregulate SOCS3, a negative regulator of the JAK/STAT pathway, leading to a decrease in PCNA expression	Breast cancer	Anti-oncogenic & Downregulated	[99]
CircFBXW7	Protein encoding	-	-	FBXW7-185aa	Bind to USP28 and inhibit function in activating c-Myc	Breast cancer	Anti-oncogenic & Downregulated	[100]
	miRNA sponge	miR-197-3p	FBXW7	FBXW7	- Upregulate FBXW7 by inhibiting its repressor, miR-197-3p - FBXW7 negatively regulates c-Myc			
CircSETD2	miRNA sponge	miR-155-5p	SCUBE2	SCUBE2	- Upregulate SCUBE2 by inhibiting its repressor, miR-155-5p - SCUBE2 negatively regulates CDK4, and cyclin D1	Breast cancer	Anti-oncogenic & Downregulated	[101]
CircZFR	Protein sponge	-	-	SSBP1	Promote the formation of CDK2/cyclin E1 complex which later induces p-Rb and releases E2F1	Cervical cancer	Oncogenic & Upregulated	[102]
	miRNA sponge	miR-147a	CACUL1	CACUL1	- Upregulate CACUL1 by inhibiting its repressor, miR-147a - CACUL1 positively regulates CDK2, CDK4, CDK6, and cyclin D1	Colorectal cancer	Oncogenic & Upregulated	[103]
Hsa_circ_0072088 (circZFR)	miRNA sponge	miR-377-5p	NOVA2	NOVA2	- Upregulate NOVA2 by inhibiting its repressor, miR-377-5p - NOVA2 positively regulates cyclin D1, and PCNA	Lung cancer	Oncogenic & Upregulated	[104]
Hsa_circ_0000326 (circTCONS_l2_00004572)	miRNA sponge	miR-338-3p	CDK4	CDK4	Upregulate CDK4 by inhibiting its repressor, miR-338-3p	Cervical cancer	Oncogenic & Upregulated	[105]
Hsa_circ_0000263 (circTCONS_00017720)	miRNA sponge	miR-150-5p	MDM4	MDM4	- Upregulate MDM4 by inhibiting its repressor, miR-150-5p - MDM4 negatively regulates p53	Cervical cancer	Oncogenic & Upregulated	[106]
Hsa_circ_0009035 (circRACGAP1)	miRNA sponge	miR-889-3p	HOXB7	HOXB7	- Upregulate HOXB7 by inhibiting its repressor, miR-889-3p - HOXB7 positively regulates PCNA	Cervical cancer	Oncogenic & Upregulated	[107]
Hsa_circ_0003221 (circPTK2)	miRNA sponge	miR-758-3p	CPEB4	CPEB4	- Upregulate CPEB4 by inhibiting its repressor, miR-758-3p - CPEB4 positively regulates cyclin D1 and PCNA	Cervical cancer	Oncogenic & Upregulated	[108]
CircESRP1	miRNA sponge	miR-634	TPD52	TPD52	- Upregulate TPD52 by inhibiting its repressor, miR-634 - TPD52 positively regulates cyclin D1	Cervical cancer	Oncogenic & Upregulated	[109]
		miR-142-3p	ARL2	ARL2	- Upregulate ARL2 by inhibiting its repressor, miR-142-3p - ARL2 positively regulates cyclin D1 and PCNA			[110]
Hsa_circ_0000285 (circHIPK3)	miRNA sponge	miR-197-3p	ELK1	ELK1	- Upregulate ELK1 by inhibiting its repressor, miR-197-3p - ELK1 positively regulates cyclin D1 and PCNA	Cervical cancer	Oncogenic & Upregulated	[111]
CircASAP1	miRNA sponge	miR-338-3p	RPP25	RPP25	- Upregulate RPP25 by inhibiting its repressor, miR-338-3p - RPP25 positively regulates cyclin D1 and PCNA	Cervical cancer	Oncogenic & Upregulated	[112]
Hsa_circ_0008934 (circASAP1)	miRNA sponge	miR-145-5p	E2F3	E2F3	Upregulate E2F3 by inhibiting its repressor, miR-145-5p	Osteosarcoma	Oncogenic & Upregulated	[113]
Hsa_circ_0008231 (circNBPF11)	miRNA sponge	miR-338-3p	ETS1	ETS1	Inhibit miR-338-3p to upregulate ETS1, a positive regulator of the JAK/STAT pathway, leading to an increase in cyclin D1 expression	Colon cancer	Oncogenic & Upregulated	[114]
CircCTIC1	Protein sponge	-	-	NURF	Promote nuclear translocation of NURF to bind to c-Myc promotor and increase c-Myc expressiom	Colon cancer	Oncogenic & Upregulated	[115]
CircCSPP1	miRNA sponge	miR-431	ROCK1	ROCK1	- Upregulate ROCK1 by inhibiting its repressor, miR-431 - ROCK1 positively regulates CDK4, cyclin D1, and p-Rb	Colon cancer	Oncogenic & Upregulated	[116]
Hsa_circ_0005615 (circNFATC3)	miRNA sponge	miR-149-5p	TNKS	TNKS	Inhibit miR-149-5p to upregulate TNKS, a positive regulator of the Wnt/β-catenin pathway, leading to an increase in cyclin D1 expression	Colorectal cancer	Oncogenic & Upregulated	[117]
CircMETTL9	miRNA sponge	miR-551b-5p	CDK6	CDK6	Upregulate CDK6 by inhibiting its repressor, miR-551b-5p	Colorectal cancer	Oncogenic & Upregulated	[118]
CircPRMT5	miRNA sponge	miR-377	E2F3	E2F3	Upregulate E2F3 by inhibiting its repressor, miR-377	Colorectal cancer	Oncogenic & Upregulated	[119]
Hsa_circ_0007142 (circDOCK1)	miRNA sponge	miR-122-5p	CDC25A	CDC25A	Upregulate CDC25A by inhibiting its repressor, miR-122-5p	Colorectal cancer	Oncogenic & Upregulated	[120]
CircCTNNA1	miRNA sponge	miR-149-5p	FOXM1	FOXM1	- Upregulate FOXM1 by inhibiting its repressor, miR-149-5p - FOXM1 is a transcription factor of cyclin B1, and cyclin D1	Colorectal cancer	Oncogenic & Upregulated	[121]
Hsa_circ_0002577 (circWDR26)	miRNA sponge	miR-197	CTNND1	CTNND1	Inhibit miR-197 to upregulate CTNND1, a positive regulator of the Wnt/β-catenin pathway, leading to an increase in cyclin D1 and c-Myc expression	Endometrial cancer	Oncogenic & Upregulated	[122]
Hsa_circ_0001610 (circTNFRSF21)	miRNA sponge	miR-139-5p	CCNB1	Cyclin B1	Upregulate cyclin B1 by inhibiting its repressor, miR-139-5p	Endometrial cancer	Oncogenic & Upregulated	[123]
CircFIG4	miRNA sponge	miR-493-5p	E2F3	E2F3	Upregulate E2F3 by inhibiting its repressor, miR-493-5p	Esophageal cancer	Oncogenic & Upregulated	[124]
CircFNDC3B	miRNA sponge	miR-214-3p	CDC25A	CDC25A	Upregulate CDC25A by inhibiting its repressor, miR-214-3p	Esophageal cancer	Oncogenic & Upregulated	[125]
Hsa_circ_00014879 (circDCAF8)	miRNA sponge	miR-519-3p	CDC25A	CDC25A	Upregulate CDC25A by inhibiting its repressor, miR-519-3p	Esophageal cancer	Oncogenic & Upregulated	[126]
Hsa_circ_0003340 (circOGDH)	miRNA sponge	miR-940	PRKAA1	PRKAA1	- Upregulate PRKAA1 by inhibiting its repressor, miR-940 - PRKAA1 positively regulates cyclin D1, and PCNA	Esophageal cancer	Oncogenic & Upregulated	[127]
Hsa_circ_0120816 (circGFPT1)	miRNA sponge	miR-1305	TXNRD1	TXNRD1	- Upregulate TXNRD1 by inhibiting its repressor, miR-1305 - TXNRD1 positively regulates cyclin B1	Esophageal cancer	Oncogenic & Upregulated	[128]
CircPGPEP1	miRNA sponge	miR-1297	E2F7	E2F7	Upregulate E2F7 by inhibiting its repressor, miR-1297	Gastric cancer	Oncogenic & Upregulated	[129]
Hsa_circ_104433	miRNA sponge	miR-497-5p	CDC25A	CDC25A	Upregulate CDC25A by inhibiting its repressor, miR-497-5p	Gastric cancer	Oncogenic & Upregulated	[130]
CircRNF111	miRNA sponge	miR-876-3p	KLF12	KLF12	- Upregulate KLF2 by inhibiting its repressor, miR-876-3p - KLF2 positively regulates cyclin D1	Gastric cancer	Oncogenic & Upregulated	[131]
Hsa_circ_0001982 (circRNF111)	miRNA sponge	miR-1205	E2F1	E2F1	Upregulate E2F1 by inhibiting its repressor, miR-1205	Glioma	Oncogenic & Upregulated	[132]
Hsa_circ_0006470 (circMFN2)	miRNA sponge	miR-1234	TP53I11	TP53I11	Inhibit miR-1234 to upregulate TP53I11, a negative regulator of the PI3K/Akt pathway, leading to an increase in CDK2 expression	Gastric cancer	Anti-oncogenic and Downregulated	[133]
CircEVI5	miRNA sponge	miR-4793-3p	FOXO1	FOXO1	- Upregulate FOXO1 by inhibiting its repressor, miR-4793-3p - FOXO1 positively regulates p21 and p27	Gastric caner	Anti-oncogenic and Downregulated	[134]
Hsa_circ_0001495 (circCCNB1)	Protein sponge	-	-	HuR	Promote stabilization of *CCND1* mRNA	Glioma	Oncogenic & Upregulated	[135]
	miRNA sponge	miR-516b-5p	CCND1	Cyclin D1	Upregulate cyclin D1 by inhibiting its repressor, miR-516b-5p			
Hsa_circ_0012129 (circB4GALT2)	miRNA sponge	miR-761	TGIF2	TGIF2	- Upregulate TGIF2 by inhibiting its repressor, miR-761 - TGIF2 positively regulates CDK2, and CDK4	Glioma	Oncogenic & Upregulated	[136]
Hsa_circ_0030018 (circPOSTN)	miRNA sponge	miR-1297	RAB21	RAB21	- Upregulate RAB21 by inhibiting its repressor, miR-1297 - RAB21 positively regulates cyclin D1, and PCNA	Glioma	Oncogenic & Upregulated	[137]
CircCDR1as	Protein sponge	-	-	p53	Prevent p53 from interacting with its negative regulator, MDM2	Glioma	Anti-oncogenic & No expression status	[138]
Hsa_circ_0036412 (circETFA)	Protein sponge	-	-	ELAVL1	Promote stabilization of GLI2, a Hedgehog regulator, to increase cyclin D1	Hepatocellular carcinoma	Oncogenic & Upregulated	[139]
Hsa_circ_0091581 (circGPC3)	miRNA sponge	miR-526b	c-Myc	c-Myc	Upregulate c-Myc by inhibiting its repressor, miR-526b	Hepatocellular carcinoma	Oncogenic & Upregulated	[140]
Hsa_circ_0091579 (circGPC3)	miRNA sponge	miR-1287	PDK2	PDK2	- Upregulate PDK2 by inhibiting its repressor, miR-1287 - PDK2 positively regulates cyclin D1	Hepatocellular carcinoma	Oncogenic & Upregulated	[141]
CircROBO1	miRNA sponge	miR-130a-5p	CCNT2	Cyclin T2	Upregulate cyclin T2 by inhibiting its repressor, miR-130a-5p	Hepatocellular carcinoma	Oncogenic & Upregulated	[142]
CircMMP9	miRNA sponge	miR-149	CCND2	Cyclin D2	Upregulate cyclin D2 by inhibiting its repressor, miR-149	Hepatocellular carcinoma	Oncogenic & Upregulated	[143]
Hsa_circ_101555	miRNA sponge	miR-145-5p	CDCA3	CDCA3	Upregulate CDCA3 by inhibiting its repressor, miR-145-5p	Hepatocellular carcinoma	Oncogenic & Upregulated	[144]
CircZNF83	miRNA sponge	miR-324-5p	CDK16	CDK16	Upregulate CDK16 by inhibiting its repressor, miR-324-5p	Hepatocellular carcinoma	Oncogenic & Upregulated	[145]
CircSLC7A11	miRNA sponge	miR-330-3p	CDK1	CDK1	Upregulate CDK1 by inhibiting its repressor, miR-330-3p	Hepatocellular carcinoma	Oncogenic & Upregulated	[146]
Hsa_circ_0005397 (circRHOT1)	miRNA sponge	miR-326	PDK2	PDK2	- Upregulate PDK2 by inhibiting its repressor, miR-326 - PDK2 positively regulates cyclin D1 and negatively regulates p21	Hepatocellular carcinoma	Oncogenic & Upregulated	[147]
CircVAMP3	Protein sponge	-	-	CAPRIN1	Block CAPRIN1 from interacting with G3BP1 to decrease c-Myc	Hepatocellular carcinoma	Anti-oncogenic & Downregulated (in tissue)	[148]
CircLARP4	miRNA sponge	miR-761	RUNX3	RUNX3	- Upregulate RUNX3 by inhibiting its repressor, miR-761 - RUNX3 positively regulates p53, and p21	Hepatocellular carcinoma	Anti-oncogenic & Downregulated	[149]
Hsa_circ_ 0000204 (circWDR37)	miRNA sponge	miR-191	KLF6	KLF6	- Upregulate KLF6 by inhibiting its repressor, miR-761 - KLF6 negatively regulates cyclin D1, cyclin D2, and c-Myc	Hepatocellular carcinoma	Anti-oncogenic & Downregulated (in tissue)	[150]
F-circBA1	miRNA sponge	miR-148b-3p	CDC25B	CDC25B	Upregulate CDC25B by inhibiting its repressor, miR-148b-3p	Leukemia	Oncogenic & No Expression Status	[28]
Hsa_circ_0005774 (circCDK1)	miRNA sponge	miR-192-5p	ULK1	ULK1	- Upregulate ULK1 by inhibiting its repressor, miR-192-5p - ULK1 positively regulates cyclin D1, and PCNA	Leukemia	Oncogenic & Upregulated	[151]
Hsa_circ_0094100 (circSFMBT2)	miRNA sponge	miR-217	ATP1B1	ATP1B1	- Upregulate ATP1B1 by inhibiting its repressor, miR-217 - ATP1B1 positively regulates cyclin D1, and PCNA	Leukemia	Oncogenic & Upregulated	[152]
CircNFIX	miRNA sponge	miR-876-3p	TRIM31	TRIM31	- Upregulate TRIM31 by inhibiting its repressor, miR-876-3p - TRIM31 positively regulates cyclin D1	Leukemia	Oncogenic & Upregulated	[153]
Hsa_circ_0000190 (circCNIH4)	miRNA sponge	miR-142-5p	CDK6	CDK6	- Upregulate CDK6 by inhibiting its repressor, miR-142-5p - Promote MAP K signaling to increase CDK1, CDK4, and p-Rb	Lung cancer	Oncogenic & Upregulated	[154]
Hsa_circ_0014235 (circS100A2)	miRNA sponge	miR-146b-5p	YAP	YAP	Inhibit miR-146b-5p to upregulate YAP, a downstream effector of the Hippo pathway, leading an increase in CDK4 expression	Lung cancer	Oncogenic & Upregulated	[155]
	miRNA sponge	miR-520a-5p	CDK4	CDK4	Upregulate CDK4 by inhibiting its repressor, miR-520a-5p			[156]
CircDENND2A	miRNA sponge	miR-34a	CCNE1	Cyclin E1	Upregulate cyclin E1 by inhibiting its repressor, miR-34a	Lung cancer	Oncogenic & Upregulated (in tissue)	[157]
Hsa_circ_0062389 (circPI4KA)	miRNA sponge	miR-103a-3p	CCNE1	Cyclin E1	Upregulate cyclin E1 by inhibiting its repressor, miR-103a-3p	Lung cancer	Oncogenic & Upregulated	[158]
Hsa_circ_0012384 (circCMPK1)	miRNA sponge	miR-302e	CCND1	Cyclin D1	Upregulate cyclin D1 by inhibiting its repressor, miR-302e	Lung cancer	Oncogenic & Upregulated	[159]
CircABCB10	miRNA sponge	miR-584-5p	E2F5	E2F5	Upregulate E2F5 by inhibiting its repressor, miR-584-5p	Lung cancer	Oncogenic & Upregulated	[160]
Hsa_circ_0001421 (circSEC31A)	miRNA sponge	miR-4677-3p	CDCA3	CDCA3	Upregulate CDCA3 by inhibiting its repressor, miR-4677-3p	Lung cancer	Oncogenic & Upregulated	[161]
CircSATB2	miRNA sponge	miR-33a-5p	E2F7	E2F7	Upregulate E2F7 by inhibiting its repressor, miR-33a-5p	Lung cancer	Oncogenic & Upregulated	[162]
CircPITX1	miRNA sponge	miR-1248	CCND2	Cyclin D2	Upregulate cyclin D2 by inhibiting its repressor, miR-1248	Lung cancer	Oncogenic & Upregulated	[163]
Hsa_circ_0006692 (circTCONS_00025531)	miRNA sponge	miR-205-5p	CDK19	CDK19	Upregulate cyclin D19 by inhibiting its repressor, miR-205-5p	Lung cancer	Oncogenic & Upregulated	[164]
CircTP63	miRNA sponge	miR-873-3p	FOXM1	FOXM1	- Upregulate FOXM1 by inhibiting its repressor, miR-873-3p - FOXM1 positively regulates cyclin B1	Lung cancer	Oncogenic & Upregulated (in tissue)	[165]
CircFOXM1	miRNA sponge	miR-614	FAM83D	FAM83D	- Upregulate FAM83D by inhibiting its repressor, miR-614 - FAM83D positively regulates cyclin D1, and cyclin E1	Lung cancer	Oncogenic & Upregulated	[166]
Hsa_circ_0046263 (circP4HB)	miRNA sponge	miR-940	NOVA2	NOVA2	- Upregulate NOVA2 by inhibiting its repressor, miR-940 - NOVA2 positively regulates cyclin D1, and PCNA	Lung cancer	Oncogenic & Upregulated	[167]
Hsa_circ_0065214 (circSCAP)	Protein sponge	-	-	SF3A3	- Induce SF3A3 degradation to increase MDM4-S - MDM4-S positively regulates p53	Lung cancer	Anti-oncogenic and Downregulated	[168]
CircNOL10	Protein sponge	-	-	SCML1	- Prevent SCML1, a transcription factor, from ubiquitination to positively regulates HN2, and HN8 - HN2 and HN8 negatively regulate CHEK2, CDC25A, and E2F1 and positively regulate p53	Lung cancer	Anti-oncogenic and Downregulated	[169]
CircFBXO7	miRNA sponge	miR-296-3p	KLF15	KLF15	- Upregulate KLF15 by inhibiting its repressor, miR-296-3p - KLF15 positively regulates p21	Lung cancer	Anti-oncogenic and Downregulated	[170]
Hsa_circ_0082835 (circEZH2)	miRNA sponge	miR-429	EZH2	EZH2	Inhibit miR-429 to upregulate EZH2, a positive regulator of the Wnt/β-catenin pathway, leading to an increase in cyclin D1 and cyclin E1 expression	Melanoma	Oncogenic & No expression status	[171]
Hsa_circ_0062270 (circCDC45)	Protein sponge	-	-	EIF4A3	Promote *CDC45* mRNA stabilization	Melanoma	Oncogenic & Upregulated	[172]
CircPSAP	miRNA sponge	miR-331-3p	HDAC4	HDAC4	- Upregulate HDAC4 by inhibiting its repressor, miR-331-3p - HDAC4 positively regulates cyclin D1	Multiple myeloma	Oncogenic & Upregulated	[173]
CircMYBL2	Protein sponge	-	-	Cyclin F, MYBL2	- Facilitate the binding between cyclin F and MYBL2 - Block MYBL2 from activating oncogenes	Multiple myeloma	Anti-oncogenic and Downregulated	[174]
Hsa_circ_0000745 (circSPECC1)	Protein sponge	-	-	HuR	Promote CCND1 mRNA stabilization	Oral squamous cell carcinoma	Oncogenic & Upregulated	[175]
	miRNA sponge	miR-488	CCND1	Cyclin D1	Upregulate cyclin D1 by inhibiting its repressor, miR-488			
CircOSBPL10	miRNA sponge	miR-299-3p	CDK6	CDK6	Upregulate CDK6 by inhibiting its repressor, miR-299-3p	Oral squamous cell carcinoma	Oncogenic & Downregulated	[176]
CircYAP	Protein sponge	-	-	CDK4	Block CDK4 from establishing cyclin D1–CDK4 complex	Oral squamous cell carcinoma	Anti-oncogenic and Downregulated	[177]
CircARF3	miRNA sponge	miR-1299	CDK6	CDK6	Upregulate CDK6 by inhibiting its repressor, miR-1299	Osteosarcoma	Oncogenic & Upregulated	[178]
Hsa_circ_0000073 (circOMA1)	miRNA sponge	miR-1252-5p	CCNE2	Cyclin E2	Upregulate cyclin E2 by inhibiting its repressor, miR-1252-5p	Osteosarcoma	Oncogenic & Upregulated	[179]
Hsa_circ_0084582 (circCHD7)	miRNA sponge	miR-485-5p	JAG1	JAG1	- Upregulate JAG1 by inhibiting its repressor, miR-485-5p - JAG1 positively regulates c-Myc	Osteosarcoma	Oncogenic & Upregulated	[180]
CircSAMD4A	miRNA sponge	miR-218-5p	KLF8	KLF8	- Upregulate KLF8 by inhibiting its repressor, miR-218-5p - KLF8 positively regulates cyclin D1 and negatively regulates p21	Osteosarcoma	Oncogenic & Upregulated	[181]
CircE2F2	Protein sponge	-	-	HuR	Promote E2F2 mRNA stabilization	Ovarian cancer	Oncogenic & Upregulated	[182]
Hsa_circ_0000714 (circAARS)	miRNA sponge	miR-370-3p	RAB17	RAB17	- Upregulate RAB17 by inhibiting its repressor, miR-370-3p - RAB17 positively regulates CDK6, and Rb	Ovarian cancer	Oncogenic & Upregulated (in paclitaxel resistance)	[183]
CircNEIL3	miRNA sponge	miR-432-5p	ADAR1	ADAR1	- Upregulate ADAR1 by inhibiting its repressor, miR-432-5p - ADAR1 positively regulates GLI1, a downstream regulator in the Hedgehog pathway - GLI1 positively regulates CDK2, CDK4, CDK6, cyclin D1, and cyclin E1	Pancreatic ductal adenocarcinoma	Oncogenic & Upregulated	[184]
CircPVRL3	miRNA sponge	miR-194-5p	SOCS2	SOCS2	Inhibit miR-194-5p to upregulate SOCS2, a negative regulator of the PI3K/Akt pathway, leading to a decrease in CDK2, CDK4, cyclin D1, and cyclin E1 expression and an increase in p21 expression	Pancreatic ductal adenocarcinoma	Anti-oncogenic and Downregulated	[185]
Hsa_circ_0057558 (circSLC39A10)		miR-206	USP33	USP33	- Upregulate USP33 by inhibiting its repressor, miR-206 - USP33 positively regulates cyclin B1, cyclin D1, and c-Myc	Prostate cancer	Oncogenic & Upregulated	[186]
CircHMGCS1	miRNA sponge	miR-205-5p	ErBB3	ErBB3	Inhibit miR-205-5p to upregulate ErBB3, a positive regulator of the PI3K/Akt pathway, leading to an increase in cyclin D1 and CDK4 expression	Prostate cancer	Oncogenic & Upregulated	[187]
CircDPP4	miRNA sponge	miR-195	CCND1	Cyclin D1	Upregulate cyclin D1 by inhibiting its repressor, miR-195	Prostate cancer	Oncogenic & Upregulated	[188]
CircEIF3I	Protein sponge	-	-	AUF1	Prevent AUF1 from inducing CCND1 mRNA degradation	Thyroid cancer	Oncogenic & Upregulated	[189]
CircPRKCI	miRNA sponge	miR-335	E2F3	E2F3	Upregulate E2F3 by inhibiting its repressor, miR-335	Thyroid cancer	Oncogenic & Upregulated	[190]
Hsa_circ_0000644 (circKIAA1199)	miRNA sponge	miR-1205	E2F3	E2F3	Upregulate E2F3 by inhibiting its repressor, miR-1205	Thyroid cancer	Oncogenic & Upregulated	[191]
CircHACE1	miRNA sponge	miR-346	TFC2L1	TFC2L1	- Upregulate TFC2L1 by inhibiting its repressor, miR-346 - TFC2L1 negatively regulates cyclin B1, and PCNA	Thyroid cancer	Anti-oncogenic and Downregulated	[192]
CircCAMSAP1	mRNA sponge	-	SERPINH1	-	Promote SERPINH1 stabilization to further increase c-Myc translation	Nasopharyngeal carcinoma	Oncogenic & Upregulated	[193]
CircFAM114A2	miRNA sponge	miR-222-3p	P27	P27	Upregulate p27 by inhibiting its repressor, miR-222-3p	Urothelial carcinoma	Anti-oncogenic and Downregulated	[194]
		miR-146a-5p	P21	P21	Upregulate p21 by inhibiting its repressor, miR-146a-5p			
